# Case report: a novel *KERA* mutation associated with cornea plana and its predicted effect on protein function

**DOI:** 10.1186/s12881-015-0179-9

**Published:** 2015-06-23

**Authors:** Laura Roos, Birgitte Bertelsen, Pernille Harris, Anette Bygum, Hanne Jensen, Karen Grønskov, Zeynep Tümer

**Affiliations:** Applied Human Molecular Genetics, Kennedy Center, Copenhagen University Hospital, Rigshospitalet, Gl. Landevej 7, Glostrup, 2600 Denmark; Department of Chemistry, Technical University of Denmark, Kemitorvet Build. 207, DK-2800 Kgs. Lyngby, Denmark; Department of Dermatology, Odense University Hospital, Odense, Denmark; Eye Clinic, Department of Ophthalmology, Copenhagen University Hospital, Glostrup, Denmark

**Keywords:** Cornea plana 2, Hypotrichosis, KERA protein, Missense mutation, Protein modelling, Leucin rich repeat domain

## Abstract

**Background:**

Cornea plana (CNA) is a hereditary congenital abnormality of the cornea characterized by reduced corneal curvature, extreme hypermetropia, corneal clouding and hazy corneal limbus. The recessive form, CNA2, is associated with homozygous or compound heterozygous mutations of the keratocan gene (*KERA*) on chromosome 12q22. To date, only nine different disease-associated *KERA* mutations, including four missense mutations, have been described.

**Case presentation:**

In this report, we present clinical data from a Turkish family with autosomal recessive cornea plana. In some of the affected individuals, hypotrichosis was found. *KERA* was screened for mutations using Sanger sequencing. We detected a novel *KERA* variant, p.(Ile225Thr), that segregates with the disease in the homozygous form. The three-dimensional structure of keratocan protein was modelled, and we showed that this missense variation is predicted to destabilize the structure of keratocan, leading to the classical ocular phenotype in the affected individuals. All the four known missense mutations, including the variation found in this family, affect the conserved residues of the leucine rich repeat domain of keratocan. These mutations are predicted to result in destabilization of the protein.

**Conclusion:**

We present the 10th pathogenic *KERA* mutation identified so far. Protein modelling is a useful tool in predicting the effect of missense mutations. This case underline the importance of the leucin rich repeat domain for the protein function, and this knowledge will ease the interpretation of future findings of mutations in these areas in other families with cornea plana.

**Electronic supplementary material:**

The online version of this article (doi:10.1186/s12881-015-0179-9) contains supplementary material, which is available to authorized users.

## Background

Cornea plana is a rare hereditary congenital abnormality of the cornea characterized by reduced corneal curvature, extreme hypermetropia, corneal clouding and hazy corneal limbus. There is an autosomal dominant (CNA1; OMIM 121400) and a more severe autosomal recessive form (CNA2; OMIM 217300) of the disorder. The recessive form is more frequently associated with additional ocular manifestations, such as iris malformation and adhesions between cornea and iris [1–4]. The gene defective in CNA1 has not been identified yet, while homozygous or compound heterozygous mutations in the keratocan gene (*KERA*) on chromosome 12q22 are shown to cause CNA2 [5]. The protein encoded by *KERA*, keratocan, is a keratan sulfate proteoglycan with a core of leucine-rich repeats (LRR), flanked by clusters of cystein, essential to maintain the three-dimensional structure of the protein. Keratocan is an extracellular matrix molecule of the corneal stroma that is important for normal cornea morphogenesis and involved in maintaining corneal transparency [6]. Studies with mice show expression of this gene during eye development and in adult mice, it is exclusively expressed in the cornea [7]. To date, only nine different disease-associated *KERA* mutations have been reported in various populations [5, 8–11]: Three nonsense mutations, four missense mutations, one splice-site mutation, and one frameshift (single-nucleotide deletion) mutation. All the reported missense mutations are within the LRR domain, which is considered to be essential for the binding between keratocan and the collagen fibrils in the corneal matrix during development. A disruption of the protein affects both the structure and the transparency of the cornea [12].

In this report, we present the clinical data and describe a new *KERA* mutation in a Turkish family with autosomal recessive cornea plana. We suggest the genetic variation to result in destabilization of the protein structure. We furthermore apply protein modelling to predict the effect of the missense mutations on the structure of keratocan.

## Case presentation

### Clinical studies

**Fig. 1:**
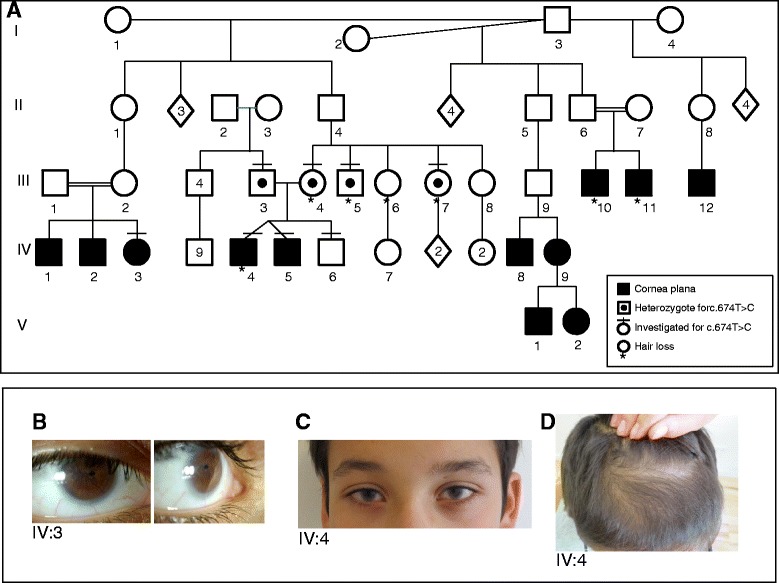
Family with cornea plana. **a.** The pedigree is consistent with autosomal recessive inheritance; **b.** The right eye of patient IV:3, with a small, malformed pupil, and a hazy corneal limbus; **c.** The eyes of patient IV:4; **d.** The scalp of patient IV:4 at age 11, showing hypotrichosis on the anterior part of vertex

A pedigree of the family is shown in Fig. 1a. The index individuals (IV:4 and IV:5) are dizygotic male twins, who were 11 years old at the time of clinical examination. They were born at gestational age 36 to healthy and unrelated parents. Birth weights were 2300 g (IV:4) and 2352 g (IV:5). Shortly after birth, IV:4 and IV:5 were found to be excessive hypermetropic (+11,75D/+11,75D and 12,75D/+13,75D) and both had corneal clouding and flat corneas. The boys have been wearing glasses since the age of 3 months, and have been monitored frequently. The clouding of the corneas decreased over the first 8 months of life. Clinical and eye examinations were carried out at the age of 10 years showing hazy corneal limbi with vessels were noticed (Fig. 1c). IV:4 has a small temporal iridocorneal synechi. Axial lengths were within normal range (A-scan using Hiscan opticon). ERG, motility, pupillary reaction, funduscopy and intraocular pressure were normal. Keratometry (Zeiss keratometer) showed bilateral flat corneas and high astigmatism in both boys (Additional file 1: Table S1).

The boys’ general development has been in the lower range of normal. They attend a normal school at normal age level, but perform in the low end of their class. They both have slight behavioural problems and motor tics, which are also found in their unaffected younger brother. From the age of 9 years IV:4 has experienced progressive frontal hair loss on vertex, the hair being lighter in colour and lusterless (Fig. 1d). Hair microscopy was normal without any signs of hair shaft disorder. IV:5 has slight general hypermobility and pes planus.

### Family history

There were several affected individuals in the family, and the pedigree was suggestive of autosomal recessive inheritance (Fig. 1a). Clinical information of the individuals III:10, III:11, IV:3, IV:9, V:1 and V:2 was extracted from charts of previous examinations. For individuals III:12, IV:1, IV:2 and IV:8 clinical information was obtained from family members. Clinical details on family members are found in Table 1, Fig. 1b and Additional file 1: Table S1.

**Table 1:** Clinical features of the affected individuals

Individual	Present age (years)	Cornea plana	Hazy corneal limbus	Irido-corneal synechi	Other eye abnormalities	Other systemic abnormalities
III:10	40	Yes	Yes	No	Corneal clouding has not decreased. Central corneal thinning, suspicion of posterior lenticonus	Hair loss in early adulthood
III:11	39	Yes	Yes	No	Corneal clouding decreased within the first year. Slight lenticonus	Hair loss in early adulthood
IV:3	22	Yes	Yes	Yes	Corneal clouding decreased within the first year. Small, eccentric malformed pupil (right) (Fig. 2a)	None
IV:4	11	Yes	Yes	Yes	Corneal clouding decreased within the first year.	Hair loss. Motor tics. Slight learning disability
IV:5	11	Yes	Yes	No	Corneal clouding decreased within the first year.	Slight learning disability. Soft cartilage, hypermobility. Motor tics
IV:9	35	Yes	No	No	Corneal clouding, anterior pole cataract, remnants of pupillary membrane	None
V:1	6	Yes	Yes	No	Corneal clouding decreased within the first year. Ectopia of pupils, left pupil irregular	None
V:2	7	Yes	No	Yes	Slight corneal clouding, decreased within the first year. Small, malformed and ectopic pupil (left)	None

### Molecular studies

The clinical features and family history prompted us to sequence *KERA.* Genomic DNA was isolated from peripheral blood lymphocytes using Chemagnic Magnetic Separation Modile 1 (Chemagen, Baesweiler, Germany). Coding exons (exon 2 and 3) of *KERA* were amplified from genomic DNA of the investigated family members using PCR. Purified PCR products were sequenced using BigDye Terminator v3.1 Cycle Sequencing Kit (Applied Biosystems, Foster City, CA). Sequencing products were purified and run on an ABI 3130 XL genetic analyzer (AppliedBiosystems). Sequences of the coding exons and 10 bp of the flanking introns were analyzed using SeqScape software (AppliedBiosystems). Primer sequences and PCR conditions are available on request.

A novel missense mutation was detected in exon 2 of *KERA*, c.674T>C, which is predicted to result in substitution of isoleucine at position 225 with threonine, p.(Ile225Thr). All affected family members investigated for the mutation (IV:3, IV:4 and IV:5) were homozygous, while the clinically unaffected parents (III: 3 and III:4), the brother (IV:6), a maternal aunt (III:7) and a maternal uncle (III:5) were all heterozygous for the mutation. . The mutation is absent from the 1000 Human Genome [13] and NHLBI Exome Sequencing [14] databases. The Ile225 lies within the LRR domain and is a highly conserved amino acid. The *in silico* prediction programs SIFT [15] and Polyphen2 [16] predict the substitution to be pathogenic (SIFT score 0.00 and Polyphen2 score 0.999).

### Protein modelling

We modelled the three-dimensional structure of the keratocan protein to predict the effect of the present mutation and the three previously published missense mutations on protein structure/function.

**Fig. 2:**
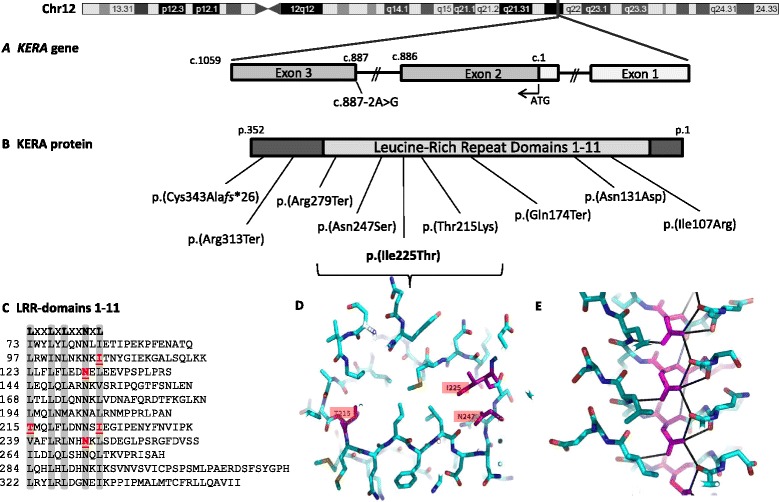
The *KERA* mutations associated with cornea plana. **a**. Schematic representation of the *KERA* gene and **b.** the predicted protein, including the positions of the disease causing mutations identified until now. The p.(Ile225Thr) missense mutation, identified in this study, changes an isoleucine (Ile, I) to a threonine (Thr, T) at amino acid position 225 (shown in bold). Seven out of nine mutations, including the p.(Ile225Thr) mutation, are predicted to affect the leucine-rich repeat region, LRR. **c.** Alignment of the leucine rich repeat domains show the 11 consensus motifs LXXLXLXXNXL (shaded with gray) and the positions of the five missense mutations p.(Ile107Arg), p.(Asn131Asp), p.(Thr215Lys), p.(Ile225Thr) and p.(Asn247Ser) that are underlined (I,N, T, I, and N, respectively). **d.** The transverse section of the protein model through the solenoid has been made around the 7th repeat and amino acids Thr215 (T215), Ile225 (I225) and Asn247 (N247) is indicated. The mutations leading to substitution of these aminoacids are shown in 2B. **e.** A side view of the structure shows the hydrogen bonding network of the asparagines which form the Asn-ladder. The structure is made from homology modelling and the side chain rotamers may be different in the human protein. The dotted lines show the hydrogen bonds

The LRR protein core of decorin (PDB entry 1xku_A) [17] was used as template. The template was chosen using BLASTp[18] to search against the protein data bank. ClustalW [19] was used to align the two sequences and SWISS-MODEL [20] was used to change the amino acids. Finally, the structure was investigated using Pymol [21]. The number of LRR repeats were predicted using LRRfinder [22]. Keratocan has a LRR structure, where the leucine rich motif is repeated several times. It has the shape of a curved solenoid, where the concave side of the structure is made up of a parallel beta-sheet and the convex side is made up of a more diverse number of structural elements (Fig. 2d and e). The leucine-rich motif LXXLXLXXNXL at the concave side of the tandem is highly conserved: The L may be leucine or a hydrophobic amino acid, N may be asparagine or cysteine, and X may be any amino acid (Fig. 2c). The interior of the LRR domain is made up of hydrophobic residues – with the exception of the conserved asparagines (Asn, N) which create an Asn-ladder stabilizing the structure as seen in Fig. 2e [23–25]. Previously, 10 LRR repeats were suggested for keratocan [5], but the LRRfinder [22] suggests presence of an 11th LRR repeat starting at amino acid position 322 (Fig. 2c).

We found that the p.(Asn131Asp) and p.(Asn247Ser) mutations affect the conserved Asn131 and Asn247 residues of the 3rd and 7th LRR-repeat. The substitution of an Asn with an Aspartic acid or a Serine will change a hydrogen bond donor to a hydrogen bond acceptor or to neutral. The important stabilizing structure of the Asn-ladder, which is seen in Fig. 2e, is likely to be destroyed.

The p.(Thr215Lys) mutation affects the Thr215 in the beginning of the 7th repeat where the threonine is pointing into the structure. Substitution of this residue with lysine is likely to destroy the structure in this area due to steric hindrance of the large lysine side chain.

The p.(Ile107Arg) and the current mutation, p.(Ile225Thr) affects the 11th residue within the 2nd and the 7th repeat (the end of the beta-strain) where the isoleucine is pointing into the structure, stabilizing the hydrophobic core. Substitution of the hydrophobic isoleucine with the hydrophilic and bulky arginine or the hydrophilic threonine will most likely destabilize the structure. It is interesting that the two mutations p.(Ile107Arg) and p.(Asn131Asp) have the same special orientation with respect to each other as the two mutations p.(Ile225Thr) and p.(Asn247Ser).

## Discussion

This family, with a novel missense mutation *KERA* [p.(Ile225Thr)], exhibits the classical ocular phenotype of autosomal recessive cornea plana, with excessive hypermetropia, hazy corneal limbus, and (although variable) iris malformation and iridocorneal adhesions.

Only nine different disease-associated mutations have been previously reported in *KERA* [4;5;8–11] (Fig. 2a, b, c). There are several lines of evidence suggesting that p.(Ile225Thr) is causative: The mutation segregates with the disease in the family and it affects a conserved residue located within the LRR domain which is predicted to be important for protein function. *In silico* analysis predicts the mutation to be damaging and protein modelling suggests that substitution of isoleucine at position 225 of the 7th LRR repeat will result in destabilization of the protein structure. We therefore consider this mutation the most likely cause of cornea plana in this family.

Three affected family members were homozygous for the mutation. The parents of patient IV:3 are consanguineous. The parents of the index patients IV:4 and IV:5 are not, to their own knowledge, consanguineous, but descend from the same area. Since they are both heterozygous for the same mutation, we speculate that they are distantly related. If the mutation is found in other Turkish families, however, a founder effect could be considered.

Several family members have sparse scalp hair, and one of the twins exhibits severe frontal hypotrichosis. Although keratocan has not been linked to abnormalities of the hair, another protein member of the small LRR proteoglycan family, called Tsukushi (TSK), is found to be expressed at the restricted areas of hair follicles during the morphogenesis, and targeted disruption of *TSK* causes the hair cycle to be delayed in mice [17]. However, the expression pattern of keratocan in the hair follicle has not been examined. Hair loss has not previously been described in connection with cornea plana. Pedigree analysis does not show a clear connection between hair loss and the mutation - family members not homozygous for the mutation also show some thinning of the hair, which might be androgenetic. If more cases of hair loss in cornea plana patients emerge, expression studies of keratocan in the hair follicle should be considered.

## Conclusions

*KERA* mutations are very rare and we present the 10th pathogenic mutation identified so far. Protein modelling is a useful tool in predicting the effect of missense mutations. Our studies suggest that the five amino acid substitutions associated with cornea plana are likely to exercise their effect by destabilizing the structure of the keratocan protein, underlining the importance of this domain for the protein function. This is valuable knowledge when interpreting the clinical significance of future *KERA* mutations.

### Consent

The project conformed to the tenets of the Declaration of Helsinki and was approved by the Regional Scientific Ethical Committee for Copenhagen, Denmark. Written informed consent was obtained from the patient for publication of this Case report and any accompanying images. A copy of the written consent is available for review by the Series Editor of this journal.

## Additional file

Additional file 1: Table S1. Best corrected visual acuity, biometric characteristics and refractive error of affected family members. Topography of patient IV:4.

## References

[1] Khan AO, Aldahmesh M, Meyer B (2006). Corneal ectasia and hydrops in a patient with autosomal recessive cornea plana. Ophthalmic Genet.

[2] Khan AO, Aldahmesh MA, Al-Gehedan S, Meyer BF, Alkuraya FS (2009). Corneal decompensation in recessive cornea plana. Ophthalmic Genet.

[3] Tahvanainen E, Forsius H, Kolehmainen J, Damsten M, Fellman J, de la Chapelle A (1996). The genetics of cornea plana congenita. J Med Genet.

[4] Khan AO, Aldahmesh M, Meyer B (2006). Recessive cornea plana in the Kingdom of Saudi Arabia. Ophthalmology.

[5] Pellegata NS, Dieguez-Lucena JL, Joensuu T, Lau S, Montgomery KT, Krahe R (2000). Mutations in KERA, encoding keratocan, cause cornea plana. Nat Genet.

[6] Tasheva ES, Funderburgh JL, Funderburgh ML, Corpuz LM, Conrad GW (1999). Structure and sequence of the gene encoding human keratocan. DNA Seq.

[7] Liu CY, Shiraishi A, Kao CW, Converse RL, Funderburgh JL, Corpuz LM (1998). The cloning of mouse keratocan cDNA and genomic DNA and the characterization of its expression during eye development. J Biol Chem.

[8] Ebenezer ND, Patel CB, Hariprasad SM, Chen LL, Patel RJ, Hardcastle AJ (2005). Clinical and molecular characterization of a family with autosomal recessive cornea plana. Arch Ophthalmol.

[9] Khan A, Al-Saif A, Kambouris M (2004). A novel KERA mutation associated with autosomal recessive cornea plana. Ophthalmic Genet.

[10] Liskova P, Hysi PG, Williams D, Ainsworth JR, Shah S, de la Chapelle A (2007). Study of p.N247S KERA mutation in a British family with cornea plana. Mol Vis.

[11] Dudakova L, Palos M, Hardcastle AJ, Liskova P (2014). Corneal endothelial findings in a Czech patient with compound heterozygous mutations in *KERA*. Ophthalmic Genet.

[12] Hassell JR, Birk DE (2010). The molecular basis of corneal transparency. Exp Eye Res.

[13] Abecasis GR, Auton A, Brooks LD, Depristo MA, Durbin RM, 1000 Genomes Project Consortium (2012). An integreated map of genetic variation from 1,092 human genomes. Nature.

[14] Tennessen JA, Bigham AW, O’connor TD, Fu W, Kenny EE, Gravel S (2010). Evolution and functional impact of rare coding variation from deep sequencing of human exomes. Science.

[15] Ng PC, Henikoff S (2001). Predicting deleterious amino acid substitutions. Genome Res.

[16] Adzhubei IA, Schmidt S, Peshkin L, Ramensky VE, Gerasimova A, Bork P (2010). A method and server for predicting damaging missense mutations. Nat Methods.

[17] Scott PG, McEwan PA, Dodd CM, Bergmann EM, Bishop PN, Bella J (2004). Crystal structure of the dimeric protein core of decorin, the archetypal small leucine-rich repeat proteoglycan. Proc Natl Acad Sci U S A.

[18] Altschul SF, Madden TL, Schaffer AA, Zhang J, Zhang Z, Miller W (1997). Gapped BLAST and PSI-BLAST: a new generation of protein database search programs. Nucleic Acids Res.

[19] Larkin MA, Blackshields G, Brown NP, Chenna R, McGettigan PA, McWilliam H (2007). Clustal W and Clustal X version 2.0. Bioinformatics.

[20] Arnold K, Bordoli L, Kopp J, Schwede T (2006). The SWISS-MODEL workspace: a web-based environment for protein structure homology modelling. Bioinformatics.

[21] The PyMOL Molecular Graphics System [computer program]. Version Version 1.5.0.4 Schrödinger, New York, USA: LLC; 2014.

[22] Offord V, Coffey TJ, Werling D (2010). LRRfinder: a web application for the identification of leucine-rich repeats and an integrative Toll-like receptor database. Dev Comp Immunol.

[23] Bella J, Hindle KL, McEwan PA, Lovell SC (2008). The leucine-rich repeat structure. Cell Mol Life Sci.

[24] Kobe B, Deisenhofer J (1994). The leucine-rich repeat: a versatile binding motif. Trends Biochem Sci.

[25] Kobe B, Deisenhofer J (1995). Proteins with leucine-rich repeats. Curr Opin Struct Biol.

