# Supplementary material for: Case report: a novel KERA mutation associated with cornea plana and its predicted effect on protein function
Source: BMC Med Genet. 2015 Jun 23;16:40. doi: 10.1186/s12881-015-0179-9 (PMC4630934; doi:10.1186/s12881-015-0179-9)

**Supplementary Material**

Best corrected visual acuity, biometric characteristics and refractive error of affected family members.

| **Indivi-dual** | **Best corrected visual acuity** | | **Keratometry (mean value in D of two measurements, 90° apart)** | | **Refractive errors (D)** | | **Axial length (mm)** | |
| --- | --- | --- | --- | --- | --- | --- | --- | --- |
|  | Right | Left | Right | Left | Right | Left | Right | Left |
| III:10 | 0,3 | 0,3 | 51.9 | 46.5 | -11 sph | -11 sph | 24,6 | 24,9 |
| III:11 | 0,3 | 0,2 | 32.8 | 32.5 | +13 sph -1,5 cyl | +14,5 sph -2 cyl | NA | NA |
| IV:3 | 0,4 | 0,2 | 32.9 | 32.9 | +9 sph -2 cyl | +9 sph -5.5cyl | NA | NA |
| IV:4 | 0,66 | 0,66 | 35.3 | 33.8 | +1.5 sph -3 cyl | +5 sph -2.5 cyl | 24 | 23,2 |
| IV:5 | 0,66 | 0,66 | 31.4 | 31.5 | +4 sph -2,5 cyl | +6,25 sph -4,5 cyl | 24,8 | 23,7 |
| IV:9 | 0,5 | 0,5 | NA | NA | +10 sph | +15 sph | NA | NA |
| V:1 | 0,66 | 0,66 | NA | NA | +10 sph -0,75 cyl | +9,5 sph -2,75 cyl | 17,67  (4 months old) | 17,57  (4 months old) |
| V:2 | 0,33 | 0,33 | 30.7 | Not possible | +3,25 sph -2,25 cyl | +18 sph -3,5 cyl | NA | NA |

NA=not available, D= diopters, sph=spherical, cyl=cylindrical

Topography of patient IV:4, showing irregular astigmatism


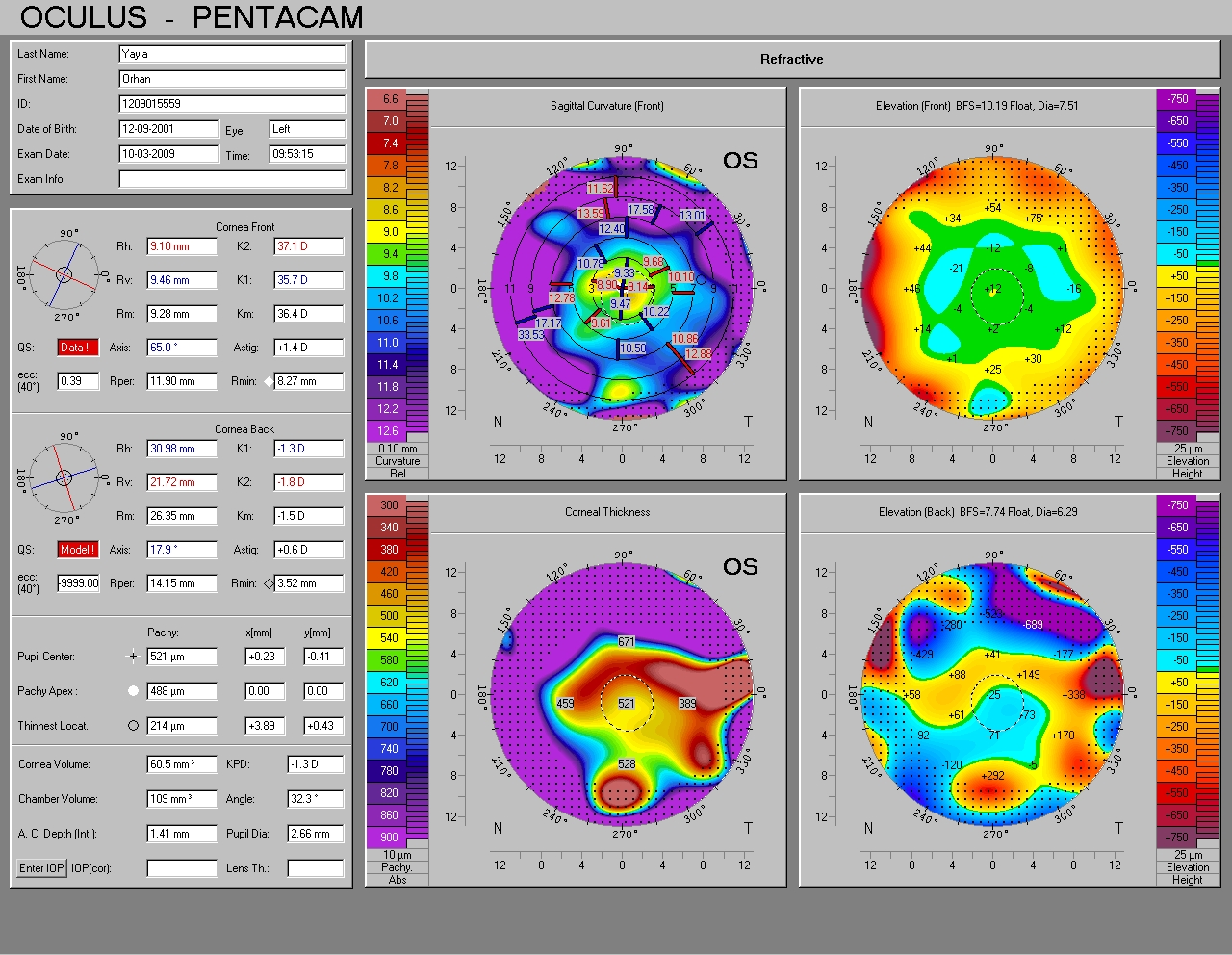


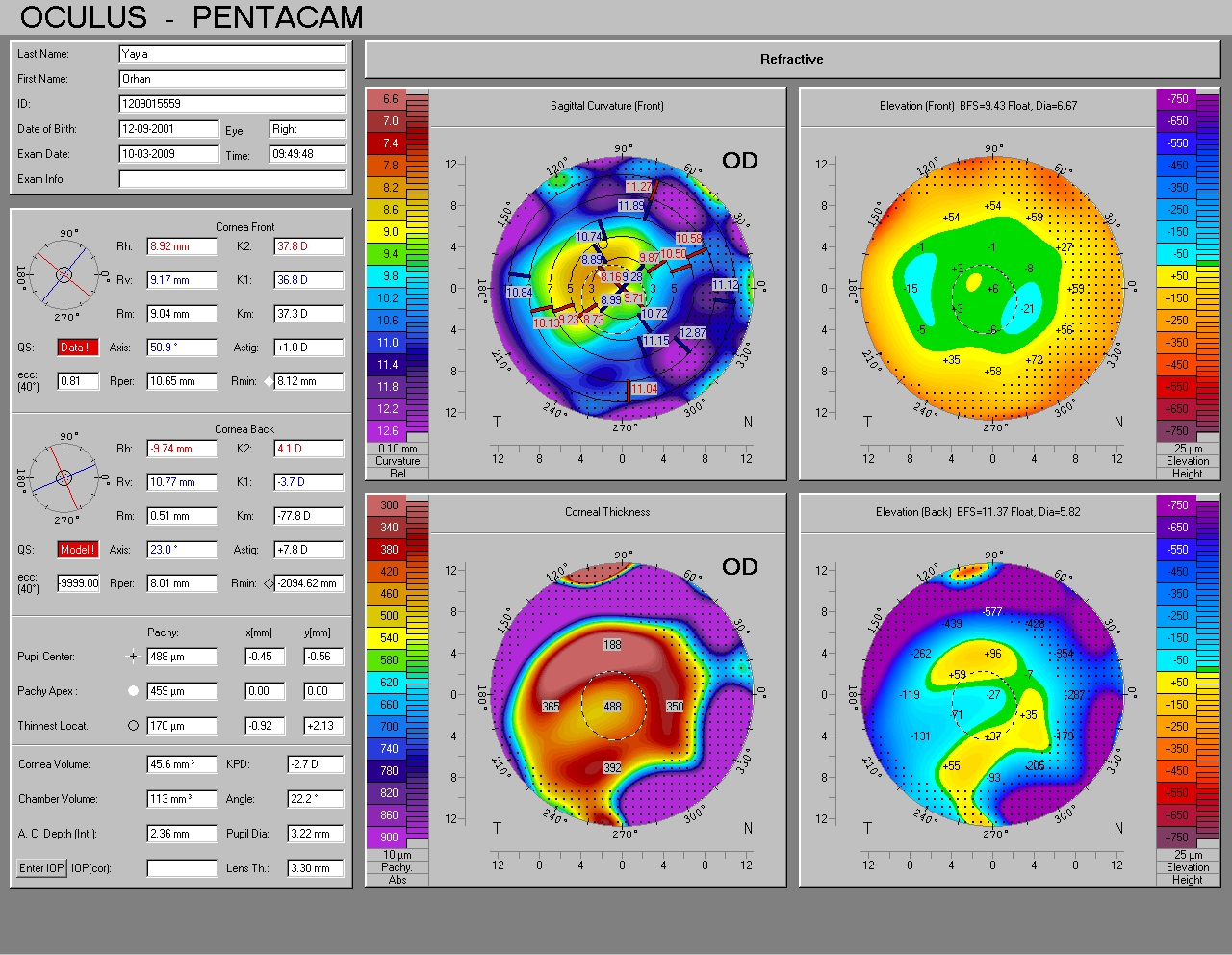

Supplement: Additional file 1: Table S1. — Best corrected visual acuity, biometric characteristics and refractive error of affected family members. Topography of patient IV:4. [file 12881_2015_179_MOESM1_ESM.docx]
